# Transitioning Adolescents to Adult HIV Care in the United States: Implementation Lessons from the *iTransition* Intervention Pilot Trial

**DOI:** 10.3390/tropicalmed9120297

**Published:** 2024-12-03

**Authors:** Amanda E. Tanner, Sulianie Mertus, Mohammed Sheikh Eldin Jibriel, Rakira Urquhart, Keenan Phillips, Nadia Dowshen, Srija Dutta, Madeleine H. Goldstein, Susan Lee, Kayla Knowles, Kaja Darien, Kelly L. Rulison, Julia Madden, Sophia A. Hussen

**Affiliations:** 1 Department of Public Health Education, University of North Carolina Greensboro, 437 Coleman, Greensboro, NC 27402, USA; smmertus@uncg.edu (S.M.); m_jibriel@uncg.edu (M.S.E.J.); rlurquhart@uncg.edu (R.U.); kaphillips@uncg.edu (K.P.); 2 Children’s Hospital of Philadelphia, 2716 South Street, Philadelphia, PA 19146, USA; dowshenn@chop.edu (N.D.); lees8@chop.edu (S.L.); knowlesk1@chop.edu (K.K.); darienk@chop.edu (K.D.); maddenjr@chop.edu (J.M.); 3 Perelman School of Medicine, University of Pennsylvania, 3400 Civic Center Boulevard, Philadelphia, PA 19104, USA; 4 Hubert Department of Global Health, Rollins School of Public Health, Emory University, 1518 Clifton Road, Atlanta, GA 30322, USA; srija.dutta@emory.edu (S.D.); sophia.ahmed.hussen@emory.edu (S.A.H.); 5 Department of Pediatrics, School of Medicine, Emory University, 100 Woodruff Circle, Atlanta, GA 30322, USA; madeleine.goldstein@emory.edu; 6 Prevention Strategies, LLC, 9 Provence Ct, Greensboro, NC 27410, USA; kelly@preventionstrategies.com; 7 Department of Medicine, School of Medicine, Emory University, 100 Woodruff Circle, Atlanta, GA 30322, USA

**Keywords:** healthcare transition, HIV, intervention, mHealth, youth and young adults

## Abstract

Although every youth in pediatric/adolescent HIV care will need to transition to adult-oriented care, there are no existing evidence-based interventions to optimize health through this process. Healthcare transition poses a persistent challenge to the health of youth living with HIV, which may result in gaps in care engagement, medication adherence, and viral suppression. Our process evaluation of *iTransition*, a multilevel mobile health (mHealth) intervention, included iterative interviews with youth, providers, and Transition Champions. These data, along with team meeting notes, highlight the important role the intervention plays in addressing healthcare transition-related challenges, positioning it to fill a critical gap for both youth and providers. It also highlights important individual (e.g., competing priorities of youth and providers), clinical (e.g., electronic health record integration), and contextual (e.g., clinical policies during COVID-19 pandemic) challenges to intervention reach and implementation. More work is needed to refine interventions to support care continuity for youth living with HIV as they transition to adult-oriented care.

## 1. Introduction

Youth living with HIV in the United States face significant barriers to care engagement that can have considerable effects on their health outcomes; recent research suggests that overall, only 55% of youth aged 13–24 years old living with HIV are engaged in HIV care and only 66% are virally suppressed [1]. These challenges are further accentuated during transition from pediatric/adolescent- to adult-oriented care [2,3]. In the United States, HIV-related healthcare transition (HCT) typically occurs around age 24 [4] and is often associated with disruptions to care retention, medication adherence, and viral suppression [5,6,7]. For instance, following expected HCT, less than 40% of transition-eligible youth are linked to adult care, and in the 1–2 years following, only 50–60% of those linked remain engaged in care [5,6,8,9]. A seamless HCT experience can aid in retention and viral suppression for youth living with HIV—with implications for individual morbidity and mortality as well as population health [10]. Despite the multilevel barriers and challenges to HCT [11], no evidence-based interventions exist to improve HCT outcomes for youth living with HIV [4,12].

To address this gap, we developed and pilot-tested *iTransition*, a multilevel mHealth intervention, to facilitate HIV-related HCT [13]. This paper describes lessons learned during *iTransition’s* implementation, offering practical insights and strategies to enhance future HIV-related HCT interventions.

## 2. Materials and Methods

### 2.1. Study Settings

We conducted the *iTransition* study at two sites in the United States. The first site was the Grady Ponce de Leon Center, a high-volume HIV care center in Atlanta, Georgia (GA), which contains both pediatric/adolescent- and adult-oriented clinic spaces in the same building but has no formal HCT protocol. The second site was the Children’s Hospital of Philadelphia (CHOP) in Philadelphia, Pennsylvania (PA). CHOP has two academic hospital-based HIV clinics that serve adolescents and young adults living with HIV; each of these adolescent clinics has a formal HCT protocol. We also enrolled providers at four of the adult clinics that often receive patients from CHOP post-HCT.

### 2.2. Intervention Overview

We describe the *iTransition* intervention research protocol elsewhere [13]. Briefly, it is a multilevel, theory-informed mHealth intervention that builds on our team’s extensive HCT work [5,6,11,14,15]. mHealth interventions have been successfully used to improve medication adherence, care retention, and viral suppression among youth living with HIV [1,2,3,4]. Building on this work, to develop *iTransition*, we collaborated with a Design Team that included pre- and post-HCT youth (N = 9) as well as pediatric/adolescent- and adult-oriented HIV care providers (N = 8) in Atlanta and Philadelphia. The resultant *iTransition* intervention includes (1) a youth-facing mobile phone application (“app”) with educational tools, medication reminders, readiness assessments, and interactive quizzes and (2) a provider-facing web-based console (separate from the electronic health records (EHRs) platform) with a dashboard including educational content, opportunities to track youth progress, and a private provider–patient chat option.

We conducted the *iTransition* pilot trial from 2020 to 2023, facing unique challenges related to trial recruitment and implementation during the COVID-19 pandemic. The trial included four participant groups: (1) historical control group, (2) youth intervention group, (3) provider intervention group, and (4) Transition Champion group (i.e., staff members from each participating clinic that supported HCT at their clinic). Research assistants obtained informed consent from all participants involved in the study. Participants received incentives for their time and effort. The study was conducted according to the guidelines of the Declaration of Helsinki, and all study protocols were approved by the Institutional Review Board (IRB) at CHOP, which served as the IRB of record for all participating institutions. The study is registered with ClinicalTrials.gov (NCT04383223).

### 2.3. Data and Analysis

Research staff recruited transition-eligible (i.e., within 12 months of anticipated HCT) youth living with HIV as well as providers from the aforementioned 3 pediatric/adolescent and 5 adult HIV clinics in Atlanta, GA, and Philadelphia, PA. As part of our process evaluation and ensure different perspectives were included, a random subset of intervention youth, providers, and Transition Champions were also invited (and all agreed) to complete qualitative interviews to examine experiences with *iTransition* use. We conducted 23 iterative semi-structured interviews (average time: 23.5 min; range 8.5–86 min) using a Social Cognitive Theory-informed interview guide with intervention youth (n = 9), providers (n = 6), and Transition Champions (n = 4) to examine intervention experiences over time, including usage, challenges, and recommendations for intervention improvement.

A professional transcription company transcribed the interview audio data. We managed the data in Atlas.ti.24 (Berlin, Germany), and a subgroup of five research team members applied a thematic analysis to organize the participants’ responses. Thematic content analysis is an approach to recognizing and establishing patterns in a data set using codes and theme categories [16]. Due to the evaluative nature of this analysis, we aimed to identify the similarities and differences within and across participant groups (e.g., youth and provider participants). We developed a preliminary codebook [17] that included pre-determined deductive codes related to the Social Cognitive Theory [5] domains of interest and inductive codes that emerged from the data. The research team coded the transcripts and resolved all discrepancies via discussion. To ensure trustworthiness of the study, our team used theory to guide our data collection and analysis and engaged in team member cross-check for data triangulation [6].

To identify HIV-related HCT intervention implementation lessons, we abstracted and analyzed data from research team meeting notes and the process evaluation interviews with intervention youth, providers, and Transition Champions.

## 3. Results

### 3.1. Participant Characteristics

Youth participants primarily identified as Black (94%), cis male (85%), and identified as a sexual minority (82%), reflecting the HIV epidemic in the United States [7]. The providers and Transition Champion participants identified as mostly white (58%) and cis female (67%); they represented all eight clinics across the two sites and various clinical roles (e.g., social worker and physician). See Table S1 for more details.

Our process evaluation identified themes affecting the implementation process related to intervention reach as well as *iTransition*-specific benefits and challenges.

### 3.2. iTransition Reach

There were several issues identified that affected the reach of *iTransition*, including participants’ overall transition knowledge and prioritization, contextual challenges, and suboptimal use of *iTransition* by both youth and providers.

Participants, especially youth, reported low knowledge about the process of transitioning to adult HIV care. A participant expressed, ‘*I’m still slightly confused if I’m transitioning, I’m still in the process of transitioning, who do I speak to, who do I go to*’ (Youth A). After discussing what to anticipate with their care team, youth felt more informed, ‘*It’s mostly been communications from my doctors or providers, just having those sessions where they told me what the process is going to look like*’ (Youth A). Overall, knowledge levels led to low prioritization of participating in the *iTransition* study. While, in general, pediatric and adolescent providers were more attuned to the importance of HCT, they still reported many barriers for their patients. One provider stated, ‘*The biggest barrier we face is having them open up to a new support team [at the adult clinic] and engaging with that support team*’ (Provider A). These challenges often meant youth looked for support from their pediatric/adolescent providers, which affected the youth’s ability to engage in adult care and meant that adult providers were less focused on supporting youth as they transitioned to adult care.

In addition, our research team meeting notes archived how contextual factors also contributed to *iTransition* recruitment challenges. For both control and intervention groups, recruitment was slower than anticipated. This slower recruitment was in part due to the specific criteria for recruitment of youth (e.g., within 12 months of transitioning to adult care) and the window in which the youth had their HIV care appointment at the pediatric/adolescent clinic. These challenges were further compounded by the COVID-19 pandemic, which changed the clinical environment, including more telehealth visits and restrictions on research activities. It was also a time of extreme challenges for many people, especially youth living with HIV, as they were managing the other challenges that the pandemic exacerbated related to financial, social, and emotional stress. These individual participant and contextual challenges affected recruitment, which also impacted other implementation issues.

### 3.3. iTransition: Benefits

Our process evaluation provided important information about the benefits and challenges to the *iTransition* intervention that have implications for usage. Both youth and providers agreed on *iTransition’s* benefits and utility in several areas. Both groups found that medication adherence was supported by the app’s reminders, which were viewed as particularly beneficial. One youth noted, ‘*Oh yeah, it’s very helpful with the alarms and stuff and the checking off which medicines you take and when, with the reminders to give you daily [medication] reminders*’ (Youth C). Providers similarly appreciated the tailored reminders, with one stating, ‘*I think a really good strength of it is the medication adherence check*’ (Provider C). *iTransition’s* organization of resources, such as testing locations and provider details, was valued for its convenience. A provider explained, ‘*You know, having all of that at their fingertips is really important, like the stuff they need, their medications*’ (Provider B). Both youth and providers also highlighted the app’s communication feature as a major strength that enabled ongoing interaction, with a provider noting, ‘*It’s a way for them to, and for me, to like communicate with folks straight on*’ (Provider A).

Youth participants acknowledged additional individual benefits. For example, one youth mentioned that *iTransition* helped make their health condition feel a bit less overwhelming, ‘*It kind of helped me, you know, personally make something that’s serious a bit, you know, lighthearted and not so intense—like, yes, I’m having to take this pill every day for the rest of my life to stay alive*’ (Youth D). This shift in tone helped alleviate some of the perceived burden and stigma associated with managing their HIV positive status.

### 3.4. iTransition: Challenges

Despite these benefits, overall *iTransition* usage levels were suboptimal for both youth and provider participants. As noted above, the timing of implementation (e.g., during the COVID-19 pandemic) affected recruitment and prioritization of healthcare behaviors for youth, including those related to HCT. Further, although *iTransition* includes many strategies to increase app use, such as frequent reminders (e.g., push notifications and direct reminders from staff), youth reported feeling overwhelmed with commitments to their employment, families, and school, so they were not able to give attention to *iTransition*. One youth highlighted the competing priorities:

*‘Honestly, for me, like, it’s just hard because I have to keep up with so many different kinds of systems and lists and things. And I tend to not look at things as much as I should because it’s, you know, it’s just adding to my overall sort of information overload and fatigue’*.

(Youth B)

Participants, both youth and providers, described other challenges associated with *iTransition*. A shared issue was remembering to use it regularly. One youth admitted, ‘*I always forget*’ (Youth E), while a provider acknowledged that because the app is ‘*not super high stake*’ (Provider D), it is easy for users to neglect it. Both groups also pointed out access challenges, such as inconsistent continuous access to a mobile device or reliable internet for using *iTransition*.

Healthcare providers and clinical staff reported additional challenges to engaging with *iTransition* related to their conflicting responsibilities. One provider stated, ‘*Adding another thing to a provider’s schedule or workload is always a challenge. Once they can fit it in, it’s easy, but starting a new initiative is always difficult*’ (Provider A). The lack of integration with EHR posed a significant barrier, making the provider-facing web console feel like an additional task outside of their usual workflow. One provider summarized,

*‘…anything outside of the EHR is complicated because we deal with all patient messages, all communications to other providers, sharing of records, all of that in the EHR, so it would be so much nicer if there was just like transition applications within it*’.

(Provider B)

Providers also expressed concerns that without EHR integration, the intervention might remain a secondary tool for youth, ‘*It’s not where their [youth] entire medical chart lives… it’s like another thing, so that might be confusing to them*’ (Provider B). These challenges highlight the need for enhancements, such as better integration with existing systems and strategies to increase user engagement.

Providers reported challenges to incorporating *iTransition* into their current transition process. One provider noted, ‘*So I mean, the reality is that we’re not, we’re not really using it. I think it’s just. We haven’t, we don’t have anything systematic for how we use iTransition*’ (Provider E). Some providers expressed confusion about the function and purpose of the intervention. For example, one provider stated,

*‘I’m still just confused with the—like how was this supposed to help patients or how was this supposed to help us to help patients in transitioning. I still can’t grasp—and again, maybe it’s user error, you know, what my purpose is on this platform’*.

(Provider F)

Our analysis further revealed that continuous training or workshops on how to use and implement *iTransition* would be helpful.

## 4. Discussion

While multiple mHealth interventions exist to improve HIV care continuum outcomes [1,2,3,4], to our knowledge, *iTransition* is the first mHealth intervention specifically designed to support HCT from pediatric/adolescent- to adult-oriented HIV care, a critical process that every young person living with HIV in the United States will need to go through [12]. The *iTransition* pilot trial results highlight the potential to support HCT by addressing the critical gaps in the HIV care continuum for youth living with HIV. Specific implementation issues to consider in HIV-related HCT intervention research to improve clinical practice and support health outcomes include (1) intervention reach, specifically recruitment (e.g., increasing clinical buy-in to support recruitment efforts regardless of societal context), (2) building on identified benefits of intervention (e.g., communication, resources that are HIV- and non-HIV-specific, reducing stigma, and centering the importance of HCT preparation and follow-up across clinical context), and (3) addressing intervention-specific challenges (e.g., potential EHR integration, expanding *iTransition* to focus on other care engagement issues).

Based on the participants’ overall assessment of *iTransition*, the intervention is a useful and needed tool for youth and providers, but it can be challenging to manage along with competing priorities and given the need to use other systems (e.g., EHR). In the future, mHealth intervention researchers and developers should consider creating a way for tools, like *iTransition*, to be accessed via the EHR system to ease utilization for both patients and providers. *iTransition* may be especially important at clinics without an existing HCT protocol; at clinics with protocols, ensuring that *iTransition* complements existing HCT processes without increasing clinical staff burden will be crucial.

This research had many strengths, including being the first to conduct a multisite pilot on an mHealth intervention to support HIV-related healthcare transition. Some limitations of the research should be considered. By design, the pilot trial included a small number of participants, and the process evaluation collected was qualitative, which may not generalize outside of this study context. Unexpectedly, the research was implemented during the COVID-19 pandemic, which certainly affected who was able and willing to participate both in terms of the extenuating personal circumstances surrounding that time and the (often overwhelming) proliferation of digital platforms introduced (e.g., to facilitate distance learning). In addition, the study sites were both well-resourced clinics in urban areas. Youth unable to participate because of COVID-19 or those living in more rural areas may face even more pronounced challenges related to HIV-related HCT. More work is needed to address the unique context of non-urban areas to ensure continuity of care for all youth living with HIV as they transition to adult care. Finally, this study was conducted prior to more universal access to long-acting injectable ART for young people; the changing treatment opportunities will likely affect how interventions address HCT for youth who are using the new delivery methods.

## 5. Conclusions

*iTransition* has the potential to address a critical gap in HIV care engagement for youth living with HIV. Overall, our process evaluation indicates feasibility of *iTransition* use if important individual and contextual factors can be addressed. Challenges encountered suggest that future efforts need to focus on refining implementation strategies and enhancing pre-implementation preparation (e.g., integration into existing systems for youth and providers) to achieve better outcomes in HIV-related HCT. Future work will need to examine different implementation strategies, at the individual and organizational level, to maximize reach and increase opportunities to support individual and community level health.

## Data Availability

The data presented in this study are available on request from the corresponding author due to participants’ health status (e.g., HIV diagnosis).

## Supplementary Materials

The following supporting information can be downloaded at https://www.mdpi.com/article/10.3390/tropicalmed9120297/s1, Table S1: Demographic characteristics of *iTransition* intervention participant groups.
