# Supplementary material for: Transitioning Adolescents to Adult HIV Care in the United States: Implementation Lessons from the iTransition Intervention Pilot Trial
Source: Trop Med Infect Dis. 2024 Dec 3;9(12):297. doi: 10.3390/tropicalmed9120297 (PMC11679769; doi:10.3390/tropicalmed9120297)
Supplement: Supplementary file 1 [file tropicalmed-09-00297-s001.zip › tropicalmed-3318585-supplementary.pdf]

Table S1. Demographic characteristics of *iTransition* intervention participant groups
[Entries = mean (range) or count (%)]

	Youth (N=33)	Provider & Transition Champions (N=24)
Age, years	24.6 (23-27)	
Race		
American Indian or Alaska Native	1 (3.0)	0 (0)
Asian or Pacific Islander	0 (0)	3 (12.5)
Black or African American	31 (93.9)	7 (29.2)
White	1 (3.0)	14 (58.3)
Ethnicity		
Hispanic/Latine	0 (0)	2 (8.3)
Non-Hispanic/Latine	33 (100)	22 (91.6)
Gender		
Cis-male	28 (84.8)	6 (33.3)
Cis-female	3 (9.1)	16 (66.7)
Trans female/trans woman/gender queer/gender non-confirming	3 (9.1)	0 (0)
Sexual orientation		
Straight	6 (18.2)	*
Gay	21 (63.6)	*
Bisexual	6 (18.2)	*

* Not asked of provider and Transition Champion participants
